# What drives repurchase retention in music training institutions? examining the roles of customer satisfaction, perceived value, and service quality

**DOI:** 10.1371/journal.pone.0312087

**Published:** 2024-12-31

**Authors:** Li Long, Liu Lijia

**Affiliations:** 1 College of Education Science, Yulin Normal University, Yulin City, Yuzhou District, Guangxi, China; 2 Faculty of Music and Performing Arts, Universiti Pendidikan Sultan Idris, Tanjong Malim, Perak, Malaysia; Al-Ahliyya Amman University, JORDAN

## Abstract

The rise of quality education has led to increased attention on music training as a vital means of enhancing personal qualities. However, with numerous music training institutions competing in the market, distinguishing oneself has become an urgent challenge. This study explores the key factors influencing customers’ willingness to renew their enrollment at music training institutions through a questionnaire survey. The questionnaire addresses various aspects, including customer expectations, perceived value, customer satisfaction, repurchase intention, teacher expectations, peer influence, service quality, and brand image. The findings reveal a significant positive correlation between customer satisfaction and repurchase intention, suggesting that enhancing customer satisfaction is crucial for promoting repurchase intention. Additionally, perceived value positively impacts both customer satisfaction and repurchase intention, indicating that customers are more inclined to repurchase when they believe the course’s value justifies the investment. Customer expectations also play a vital role, positively affecting perceived value and overall customer satisfaction. Furthermore, service quality and brand image significantly influence both customer satisfaction and repurchase intention, emphasizing the importance of high-quality service and a positive brand image in customer decision-making. Although the direct impact of teacher expectations on customer satisfaction is relatively weak, improving interactions between teachers and students, along with providing personalized guidance, can indirectly enhance satisfaction. Lastly, the anticipated positive correlation between peer influence and perceived value was not supported, suggesting that the impact of classmates may be less direct compared to that of teacher expectations and service quality.

## 1 Introduction

Art education is a vital component of quality education. It enhances students’ personal qualities, including moral and intellectual traits, and helps shape their unique personalities. By engaging in artistic activities such as writing, visual arts, dance, and music, students pursue comprehensive development and enhance their overall quality. Art education has become central to aesthetic education. In recent years, China has placed greater emphasis on artistic quality education, leading to the flourishing of the art training industry. Numerous training institutions have emerged, creating fierce competition. Concurrently, various media variety shows have vigorously promoted music projects, receiving positive reception. Many individuals pursue art and beauty to enhance personal value while seeking relaxation. The domestic music training market has a diverse target audience, resulting in a substantial market size. The market has surpassed 30 billion yuan and is growing at nearly 25% annually as of 2022. Furthermore, it is projected to reach 70 billion yuan by 2027 (source: IFPI Global Music Report). Among them, trainees under 18 exceed 200 million. Despite declining population growth, the training-age population is expected to remain at 250 million within five years. The 2023 Global Music Report indicates that by the end of 2022, revenue from subscribed audio streaming rose by 10.3% to 12.7 billion, with paid users reaching 589 million. Total revenue from streaming media, including subscriptions and advertising, increased by 11.5% year-on-year, totaling 17.5 billion and accounting for 67% of global recorded music revenue. Other income sources also grew, with physical sales up by 4%, performance rights revenue increasing by 8.6% to pre-pandemic levels, and synchronous revenue rising by 22.3% (source: IFPI Global Music Report). The global music streaming market is projected to grow at a compound annual growth rate of 15.67% from 2022 to 2027, reaching 31.1 billion (source: Music Streaming Market Size). In this intense market competition, it is crucial to explore how music training institutions can overcome challenges, particularly by focusing on the key factors influencing customers’ repeat purchase intentions.

The concept of repurchase intention (RI) has been studied extensively over the past decades across both academic and practical fields. Dado et al. (2012) pointed out that repurchase intention originated from research on customer satisfaction in the enterprise field, which is showing an upward trend in the education field [1]. Yet, Many art training institutions in recent years have closed or even gone bankrupt, and some have been reported by the media for taking away tuition fees [2]. Therefore, understanding the various variables that affect customers’ repeat purchase intentions is particularly urgent. It is especially important. Svoboda et al. (2013) believed that art training institutions in the market are mixed and have varying levels of expertise. Choosing a professional and reliable training institution is the most concerning issue for every customer, affecting their repurchase intention [3]. Even though a few prior studies did attempt to explain the customer repurchase intention of music training institutions by focusing on children’s experiential classes and targeting parents’ expectations, the target population of music training institutions currently spans a large age range, and research not only focuses on children, but also on adults, which is an important target group. Research should not only focus on children, but also on adults, an important target group. Additionally, the urgency of variables such as perceived value, customer expectations, and teacher expectations is particularly prominent in this context, as they directly affect customer satisfaction and repurchase decisions. However, many institutions’ children’s music programs have not achieved the expected results. To address this gap in understanding, this study will be based on expectation confirmation theory, to explore the factors that influence customers’ purchasing intentions in music training institutions, in order to enhance customer satisfaction with music courses, thereby promoting customers’ repurchase behavior. Therefore, this study will delve into the relationships among these variables, to construct a comprehensive research framework, to address current market challenges.

After the implementation of the “double reduction” policy, parents hope that their children can use their spare time to quickly learn a good amateur art skill and send their children to good training institutions (Double reduction: Reduce the homework burden of students in compulsory education and the burden of off-campus training). Music training institutions are facing intense competition due to the emergence of numerous competitors in the market; therefore, it is crucial for these institutions to adjust their marketing strategies to differentiate themselves in a crowded online environment. This research investigates customers’ demand for music education, with Hidayati et al. (2020) analyzing the factors influencing customer repurchase of music courses and their interrelationships, aiming to inform the marketing strategies of training institutions and increase customer repurchase frequency [4]. Theoretically, music training institutions can enhance customer repurchase rates by analyzing customer loyalty theory, the service quality model, relationship marketing theory, and differentiation strategies, all of which highlight the importance of service reliability, responsiveness, personalized attention, and uniqueness. Practically, the findings provide a novel perspective on repurchase intentions in music training institutions, an area that remains underexplored in the existing literature. To stand out in a fiercely competitive market, training institutions should improve teaching quality, respond swiftly to customer needs, establish long-term relationships, and innovate course content and teaching methods to enhance customer satisfaction and loyalty.

## 2 Theoretical background

### 2.1 Concept of repurchase intention

Customer repurchase intention refers to a customer’s determination to make a subsequent purchase based on their current consumption experience and interactions with existing service or goods providers, fundamentally relating to consumer loyalty. From a psychological perspective, intention represents a state of readiness for behavior, reflecting an individual’s tendency to respond to various stimuli. Miao et al. (2022) noted that repurchase simply refers to consumers buying the same brand they have previously experienced [5]. Various scholars have offered relevant definitions across different research fields. For instance, Yim et al. (1999) described repurchase intention as the tendency of customers to continue cooperation with their current transaction providers [6]. Mpinganjira (2014) defined Hema Fresh as the subjective willingness of early customers to purchase its products and actively recommend them to others [7]. Shi et al. (2012) posited that repurchase intention reflects customers’ willingness to purchase and engage with products or services, deriving from their actual experiences, which serve as reliable predictive signals [8]. Hsu et al. (2015) demonstrated that factors such as appearance, brand image, price, service quality, and safety indirectly enhance the repurchase intention of Vietnamese online travel agency websites through value perception, while website usability has a direct effect [9]. Additionally, Chiu et al. (2014) found that trust and customer satisfaction significantly influence repurchase intention via structural equation modeling [10]. Chiu et al. (2012) utilized partial least squares to analyze the Jakarta e-commerce industry, identifying perceived ease of use, customer satisfaction, and trust as key factors affecting repurchase intention [11]. Finally, Phan et al. (2023) employed the Analytic Hierarchy Process and Partial Least Squares to analyze customer repurchase intention data for online grocery retail products, revealing that the critical factors influencing repurchase intention include online store reputation, perceived quality, and perceived price [12].

In summary, this article explores customer repurchase intention in music training institutions based on scholarly research and practical considerations. After enrolling, music students receive instruction from their teachers. Their experiences during these lessons influence their tendency to repurchase courses at the institution before their current ones expire.

### 2.2 Expectancy confirmation theory

The Expectation Confirmation Theory (ECT), proposed by Chou et al. (2010), was originally applied in traditional business contexts. It posits that the gap between consumer expectations and actual experiences leads to varying levels of confirmation, which in turn affects purchase intention through satisfaction as a mediator [13]. In the field of education, Liao et al. (2017) found that students’ expectations and beliefs regarding a specific subject are positively correlated with their academic performance, including performance in that subject [14], and Rachmatullah et al. (2021) observed a similar correlation with course completion [15]. Mih et al. (2020) indicated a negative correlation between students’ expectations in a subject and test anxiety, suggesting that students who feel capable in a particular subject are less likely to experience test anxiety [16]. Furthermore, Lin et al. (2016) identified a positive correlation between expected value beliefs and various adaptive behaviors, including flow experience [17], while Xu (2020) linked these beliefs to effort and homework completion [18], and Ye et al. (2022) highlighted their relationship with perseverance [19].

In the context of music education, expectation refers to customers’ anticipated feelings and outcomes regarding music training courses, while confirmation is the evaluation of these expectations after their experiences. Satisfaction denotes the overall contentment customers derive from the course experience and plays a crucial role in driving their repurchase intentions. Researchers have explored the expected value beliefs of students in music and other subjects [20]. Other studies include students’ career intentions in music [21], factors influencing sustained involvement in music [22], and parental influence on students’ music learning [19]. Importantly, expectation in music training courses is defined as customers’ anticipated feelings and outcomes regarding the course, while confirmation is the assessment of these expectations following their experiences. Satisfaction refers to the overall level of contentment customers experience with the course, and this construct is pivotal in influencing their repurchase intentions. This study introduces peer influence, defined as the reciprocal effects among classmates, which significantly impacts students’ learning attitudes and behaviors in educational settings. Research shows that interactions among classmates affect emotions and directly influence learning experiences. By examining this construct, the aim is to explore how peer influence affects students’ perceived value and satisfaction, further impacting their willingness to repurchase music training courses. In the context of music training courses, classmates’ feedback and interactions can enhance the overall experience and satisfaction with the course.

## 3 Hypotheses

### 3.1 Customer satisfaction and repurchase intention

Dong et al. (2022) introduced the concept of customer satisfaction in marketing, suggesting that increasing customer satisfaction encourages customers to continue making purchasing decisions. This repurchase behavior, however, may not extend to other products and services [23]. Lim et al. (2020) supported that customer satisfaction arises from comparing the costs of purchasing a product or service with the benefits derived from it [24]. Wang (2012) posited that customer satisfaction is a psychological state resulting from the alignment of customers’ expectations, formed based on their consumption experiences, with their actual experiences. Customers create symbolic meanings for products based on their prior consumption knowledge and experiences [25]. McNeill et al. (2014) proposed that customer satisfaction enhances favorability toward specific products or services, increasing the likelihood of repurchase intentions [26]. Therefore, the following hypothesis is proposed.

H1: Customer satisfaction positively correlates with repurchase intention.

### 3.2 Perceived value and customer satisfaction or repurchase intention

Nitiwanakul (2021) demonstrated that customers consider the value of a product or service during the consumption process when deciding whether to make a purchase [27]. Samudro et al. (2020) elaborated on the concept of perceived value, defining it as the customer’s overall assessment of a product’s utility based on what they receive compared to what they give up [28]. Zanon et al. (2020) argued that value is an abstract concept and that perceived value is subjective, as not every customer shares the same feelings or ideas [29]. Furthermore, customers consider not only financial expenses but also the time and energy they invest in the consumption process. Additionally, while comparing their gains and losses, customers assess the net benefit they receive. Consequently, customers decide whether to make a purchase based on these three factors. Kala et al. (2024) also posited that perceived customer value is a significant factor influencing customer satisfaction and directly affects repurchase intentions [30]. Therefore, the following hypotheses are proposed.

H2: Perceived value positively correlates with customer satisfaction.
H3: Perceived value positively correlates with repurchase intention.

### 3.3 Customer expectations and perceived value or customer satisfaction

Santos et al. (2003) uncovered how customer expectations form or change as they access information about the enterprise and its products or services. Customer expectations are shaped by past purchasing experiences, feedback from friends and peers, and information and promises from marketers and competitors [31]. Qazi et al. (2017) argued that customer purchase intentions shape expectations, which can, in turn, influence the likelihood of making a purchase [32]. After making a purchase, customers evaluate the goods or services, developing a fundamental understanding of their value. Grazzini et al. (2023) indicated that a significant gap between product or service experiences and expectations leads to uncertainty or dissatisfaction [33]. When the product or service experience exceeds expectations, it generates confirmation cognition, which influences customers’ evaluations of the product based on their confirmation or non-confirmation behaviors. Moreover, customer satisfaction impacts the willingness to continue using the product and to make repeat purchases. Higher customer satisfaction is associated with a greater likelihood of repeat purchases. Therefore, customer expectations are included in the research framework as a moderating factor, leading to the following hypotheses.

H4: Customer expectations positively correlates with perceived value.
H5: Customer expectations positively correlates with customer satisfaction.

### 3.4 Teacher’s expectations and perceived value or customer satisfaction

The development of expected value theory has progressed through multiple stages, initially rooted in psychological research and gradually expanding into the field of education, where it merges with teacher expectation effects to create a theoretical system with practical applications. Friedrich et al. (2015) argued that the teacher expectation effect—also known as the Pygmalion or Rosenthal effect—refers to the positive influence of teacher expectations on students’ academic performance, behavior, psychological development, and personality traits [34]. This effect arises from the trust and expectations that teachers place in their students. Szumski et al. (2019) noted that teachers convey these expectations through their words, actions, attitudes, and behaviors, which stimulate students’ intrinsic motivation and encourage them to meet those expectations [35]. When teachers maintain high expectations for their students, they tend to focus more on their learning processes and provide additional guidance and support, which can lead to improved academic performance (Rubie Davies et al. (2020) [36]). The teacher expectation effect also serves as a form of emotional support, making students feel cared for and valued, which enhances their perceived value of the educational experience (Jiang et al. (2022) [37]). In educational practice, teachers in music training institutions should leverage the positive effects of teacher expectations. Liu (2022) emphasized that clearly expressing expectations, providing personalized guidance, and focusing on student growth can enhance curriculum satisfaction [38]. Therefore, the following hypotheses are proposed.

H6: Teacher’s expectations positively correlate with perceived value.
H7: Teacher’s expectations positively correlate with customer satisfaction.

### 3.5 Classmate’s influence and perceived value

Jansen et al. (2022) defined the influence of classmates as the mutual impact among members within the same class [39]. Individuals in peer groups often compare themselves to one another. Consequently, this influence can be either negative or positive (Lee et al., 2022) [40]. Madtha et al. (2023) indicated that consumers with limited understanding of a new brand, product, or service are more susceptible to peer influence [41]. Munoz-Carril et al. (2021) noted that peer influence can significantly enhance individuals’ perceived value of products or services [42]. This influence is evident not only through direct communication but also by subtly affecting individuals’ decisions based on their observations of peers’ consumption behaviors and attitudes [43]. Nguyen et al. (2024) found that individuals in a positive peer environment often experience a significant increase in perceived value, providing further support for our hypothesis [44]. Therefore, we formulate the following hypothesis.

H8: Classmate’s influence positively correlates with perceived value.

### 3.6 Service quality and perceived value or customer satisfaction

Ameen et al. (2020) revised the D M model by incorporating service quality variables. Research within this model demonstrates that service quality significantly impacts customer satisfaction [45]. In their study on the post-purchase intentions of value-added mobile services, Gurendrawati et al. (2022) confirmed that service quality positively affects customer satisfaction [46]. The music education institutions examined in this article function as platforms, with music experience courses serving as the corresponding products. Given that music training institutions primarily operate within the service industry, factors such as reputation, learning environment, course pricing, and service quality significantly influence the satisfaction of Latin dance students. Following the course, Bouhlel et al. (2023) concluded that customers perceive a relative value for service quality, which positively impacts their satisfaction [47]. Therefore, the following hypotheses are proposed.

H9: Service quality positively correlates with perceived value.
H10: Service quality positively correlates with customer satisfaction.

### 3.7 Brand image and customer satisfaction or repurchase intention

Brand image refers to the associations that customers form in their memory regarding an organization, which can influence their expectations and satisfaction judgments. Attitude is a predictor of behavioral intention, including purchase intention [48]. Numerous studies indicate that a positive brand image fosters customer satisfaction and loyalty [49]. Tang et al. (2020) proposed that a strong brand image can influence customer choices, patronage, and willingness to renew services [50]. Thus, brand image is crucial for establishing differentiation among stores. Afandi et al. (2023) found that brand image directly impacts customer loyalty in their empirical study of Taiwanese retail enterprises, with customer satisfaction acting as a mediator [51]. A study on Swiss retail stores examined the relationship between retail brand image, customer satisfaction, and loyalty, concluding that the impact of brand image on customer loyalty occurs through the full mediation of customer satisfaction. Brand image encompasses the totality of customers’ perceptions and associations with various brand elements. Customer satisfaction reflects customers’ perceptions of how well a company meets their expectations regarding brand performance, while customer loyalty denotes psychological approval and the intention to act in favor of a specific brand. Consequently, the better the customer’s evaluation of the retail brand image, the more the brand meets their expectations, resulting in higher satisfaction and a greater willingness to continue patronizing the brand. Therefore, the following hypotheses are proposed.

H11: Brand image positively correlates with customer satisfaction.
H12: Brand image positively correlates with repurchase intention.

### 3.8 Conceptual model

**Fig 1:**
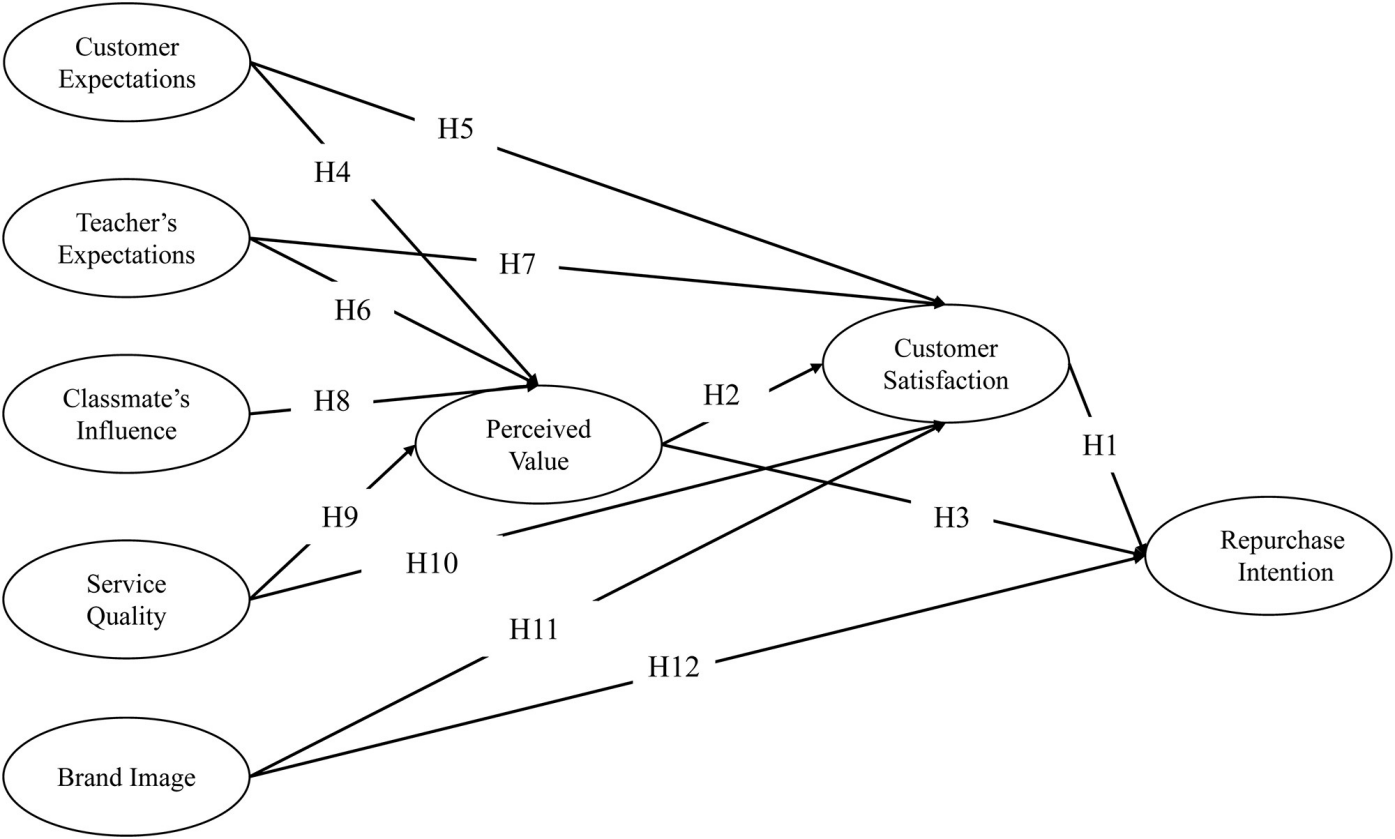
Research model.

The conceptual model is shown as Fig 1:

## 4 Experimental results

### 4.1 Source of questionnaire questions

Music training is a vital form of education that enhances students’ artistic literacy and significantly influences their learning motivation, emotional attitudes, and consumption behaviors. With society placing greater emphasis on arts education, the number of music training institutions has rapidly increased, resulting in intensified market competition. This study’s questionnaire is designed to explore the impact of music training on customer behavior. The questionnaire consists of three main parts that address key aspects of customer behavior. The first part focuses on customers’ direct experiences and expectations, including customer expectations, perceived value, and customer satisfaction. The second part addresses external factors influencing customer experience and behavior, such as teacher expectations, peer influence, and service quality. The final part examines behavioral intentions, including brand image and the willingness to repurchase. To ensure the validity of the questionnaire, measurement items were developed based on previous academic research, as shown in Table 1. Their scientific and practical validity was rigorously verified. The survey was conducted from March 4 to April 15, 2024, in music training institutions across Guangxi. This study collected a total of 305 valid questionnaires. The study’s ethics committee reviewed and waived additional consent requirements to ensure ethical compliance and data reliability.

**Table 1:** Sources of measurement items in the questionnaire.

Constructs	Sources
Customer Expectations	Qazi et al. (2017)[32]; Grazzini et al. (2023)[33]
Teacher’s Expectations	Szumski et al. (2019)[35]; Rubie-Davies et al. (2020)[36]
Classmate’s Influence	Chiu et al. (2012)[11]; Phan et al. (2023)[12]
Service Quality	Ameen et al. (2020)[45]; Hidayati et al. (2020)[4]
Brand Image	Lin et al. (2018)[49]; Tang et al. (2020)[50]
Perceived Value	Nitiwanakul (2021)[27]; Miao et al. (2022)[5]
Customer Satisfaction	Lim et al. (2020)[24]; Miao et al. (2022)[5]
Repurchase Intent	Liao et al. (2017)[14]; Miao et al. (2022)[5]

### 4.2 Factor analysis

#### 4.2.1 Reliability and validity analysis

In this experiment, we employed the KMO test to assess the applicability of the data and the feasibility of factor analysis. Additionally, we evaluated the internal consistency of the questionnaire by calculating the Cronbach’s Alpha value, which verifies its reliability and effectiveness. As shown in Table 2, the KMO sampling appropriateness is 0.876. This indicates a low partial correlation between variables and a high shared common variance, confirming its suitability for factor analysis. The Cronbach’s Alpha value of 0.903 significantly exceeds the standard threshold of 0.7, suggesting that all 31 questions in this questionnaire exhibit high internal consistency. Therefore, we conclude that the questionnaire demonstrates good stability and reliability.

**Table 2:** Reliability and effectiveness: The reliability and validity of the dataset were thoroughly assessed to determine the internal consistency of the questionnaire and to evaluate its suitability for factor analysis.

Variables	Items	Alpha	KMO
Customer Expectations	4	0.844	0.820
Teacher’s Expectations	3	0.810	0.716
Classmate’s Influence	5	0.877	0.879
Service Quality	4	0.818	0.806
Brand Image	5	0.877	0.872
Perceived Value	4	0.862	0.819
Customer Satisfaction	3	0.819	0.718
Repurchase Intent	4	0.806	0.709
All items	32	0.903	0.876

#### 4.2.2 Factor analysis

Factor analysis revealed eight principal components. As shown in Table 3, the first principal component has an initial eigenvalue of 8.084, explaining 26.077% of the total variance, with a cumulative contribution rate also at 26.077%. The second principal component has an eigenvalue of 3.009, which accounts for an additional 9.705% of the variance, raising the cumulative contribution rate to 35.782%. The third component follows closely with an eigenvalue of 2.644, contributing 8.528% to the variance and increasing the cumulative rate to 44.310%. The eigenvalues for the fourth to eighth components are 2.041, 1.793, 1.619, 1.331, and 1.184, which explain 6.584%, 5.784%, 5.224%, 4.295%, and 3.818% of the variance, respectively. Consequently, the cumulative explanatory rates rise to 50.894%, 56.678%, 61.902%, 66.197%, and 70.015%. This coverage captures the majority of the essential information in the original dataset, laying a solid foundation for further interpretation and application. It effectively demonstrates the explanatory power of our experiment and the contribution of each factor to overall data variability.

**Table 3:** Total variance explained by principal components: This section focuses on dimensionality reduction and the explanation of the dataset’s structure to extract key informational components.

Factor	Before Rotation			After Rotation		
	Eigenvalue	Variance Explained %	Cumulative %	Eigenvalue	Variance Explained %	Cumulative %
1	8.084	26.077	26.077	3.487	11.249	11.249
2	3.009	9.705	35.782	3.418	11.027	22.276
3	2.644	8.528	44.310	2.927	9.443	31.719
4	2.041	6.584	50.894	2.754	8.883	40.602
5	1.793	5.784	56.678	2.681	8.648	49.250
6	1.619	5.224	61.902	2.195	7.080	56.330
7	1.331	4.295	66.197	2.170	6.999	63.328
8	1.184	3.818	70.015	2.073	6.687	70.015

### 4.3 Confirmatory analysis

#### 4.3.1 Model indicator analysis

We conducted a confirmatory factor analysis (CFA) to assess the fit of the model. This analysis utilizes model fitting indices to explore the interrelationships and underlying structures among multiple variables. We employed the Default model, which evaluates parameters through the maximum likelihood estimation method, involving a total of 126 parameters. According to the fitting index results in Table 4, the Chi-square value is 504.660, with 412 degrees of freedom and a significance level of 0.001. This indicates a good fit between the model and the data. Additionally, the ratio of CMIN/DF is 1.225, significantly lower than the conventional acceptance standard of 3, reflecting a very good fit of the model. The comparison indices, including NFI, RFI, IFI, TLI, and CFI, are 0.893, 0.879, 0.978, 0.975, and 0.978, respectively. These values are close to or exceed the ideal standard of 0.9, indicating high fitting quality. The GFI index is 0.906, further confirming the good consistency between the model and observed data. The RMSEA value is 0.027, well below the conventional threshold of 0.08, indicating a small model error and confirming the excellent fitting effect. Overall, the model demonstrates high consistency with the actual data regarding structural design and parameter estimation, effectively explaining the complex relationships between variables.

**Table 4:** Model fit of validation factor analysis. This analysis tests the relationships between observed variables and latent variables. It evaluates the degree of fit between actual data and theoretical models by comparing various fit indices.

Model Fit	CMIN	DF	CMIN/DF	NFI	RFI	IFI	TLI	CFI	GFI	RMSEA
Fit Results	504.660	412	1.225	0.893	0.879	0.978	0.975	0.978	0.906	0.027
Judgment Std.			<3	>0.9	>0.9	>0.9	>0.9	>0.9	>0.9	<0.08

#### 4.3.2 Convergence validity analysis

To demonstrate measurement validity and internal consistency, we conducted a detailed analysis of average variance extracted (AVE) and composite reliability (CR). These analyses comprehensively evaluated the measurement validity and internal consistency of the constructs. The results, presented in Table 5, indicate that all AVE values exceed the recommended threshold of 0.5. This confirms that most observed variances are driven by latent constructs rather than measurement errors, providing strong support for the model’s structural validation. Additionally, the CR values for each construct generally exceed the standard of 0.7, indicating a high degree of internal consistency. Notably, the CR value for Brand Image reaches 0.89, demonstrating its stability and reliability within the model. This high composite reliability reveals statistical coordination and consistency among various indicators, supporting the measurement of the same concept. These analyses not only confirm the measurement reliability of the model’s constructs but also emphasize their independence, which is crucial for understanding the complex relationships among variables. The high AVE and CR values enhance the model’s internal consistency and provide robust evidence of the effective correlation between its components, thereby confirming the model’s convergence validity.

**Table 5:** Convergence validity analysis results.

Measure	Customer	Teacher’s	Classmate’s	Service	Brand	Perceived	Customer	Repurchase
	Expectations	Expectations	Influence	Quality	Image	Value	Satisfaction	Intention
**AVE**	0.60	0.58	0.62	0.57	0.65	0.60	0.55	0.59
**CR**	0.85	0.83	0.87	0.84	0.89	0.86	0.82	0.85

### 4.4 Structural equation model path analysis

This study examines the factors influencing customer satisfaction and repurchase intention in music training institutions. We assess these factors using exploratory factor analysis, confirmatory factor analysis, and structural equation modeling. As shown in Table 6, which includes unstandardized and standardized regression coefficients, standard errors, C.R., and p-values, we successfully validated the hypothesized paths in the model. Significant p-values (p < 0.05) for most paths confirm their statistical significance. However, the paths from perceived value to customer expectations and classmate influence were not supported, with p-values of 0.098 and 0.107, respectively, indicating no significant effect. In contrast, the dependence of perceived value on teacher expectations and service quality was significantly supported, with p-values of 0.018 and < 0.001, and standardized path coefficients of 0.182 and 0.286. This indicates that teacher expectations and service quality have a strong positive effect on perceived value. Additionally, customer satisfaction positively influences perceived value, supported by significant paths with p-values less than 0.05. Customer expectations emerged as the strongest predictor of customer satisfaction, with a standardized path coefficient of 0.348. However, the path from customer satisfaction to teacher expectations was not supported, with a p-value of 0.984, indicating an extremely weak effect. Furthermore, the paths from brand image, perceived value, and customer satisfaction to repurchase intention were statistically supported, demonstrating a clear positive effect. This analysis confirms direct dependencies among certain variables in the model, highlights the internal consistency of the model, and emphasizes the plausibility of the relationships between these variables. The dynamic interactions among the different potential variables in the model are also illustrated.

**Table 6:** Structural equation model path coefficient test.

Path	Unstd. Coef.	Std. Error	C.R.	P-Value	Std. Coef.	Conclusion
Perceived Value ← Customer Expectations	0.115	0.070	1.653	0.098	0.121	Not Supported
Perceived Value ← Teacher’s Expectations	0.185	0.079	2.360	0.018	0.182	Supported
Perceived Value ← Classmate’s Influence	0.097	0.060	1.610	0.107	0.101	Not Supported
Perceived Value ← Service Quality	0.333	0.096	3.454	<0.001***	0.286	Supported
Customer Satisfaction ← Customer Expectations	0.319	0.070	4.580	<0.001***	0.348	Supported
Customer Satisfaction ← Teacher’s Expectations	0.001	0.073	0.020	0.984	0.002	Not Supported
Customer Satisfaction ← Service Quality	0.225	0.089	2.525	0.012	0.200	Supported
Customer Satisfaction ← Brand Image	0.157	0.059	2.655	0.008	0.175	Supported
Customer Satisfaction ← Perceived Value	0.194	0.065	2.976	0.003	0.201	Supported
Repurchase Intention ← Brand Image	0.152	0.068	2.237	0.025	0.162	Supported
Repurchase Intention ← Perceived Value	0.187	0.076	2.459	0.014	0.185	Supported
Repurchase Intention ← Customer Satisfaction	0.224	0.089	2.522	0.012	0.213	Supported

* p<0.05;

** p<0.01;

*** p<0.001.

### 4.5 Discussion

**Fig 2:**
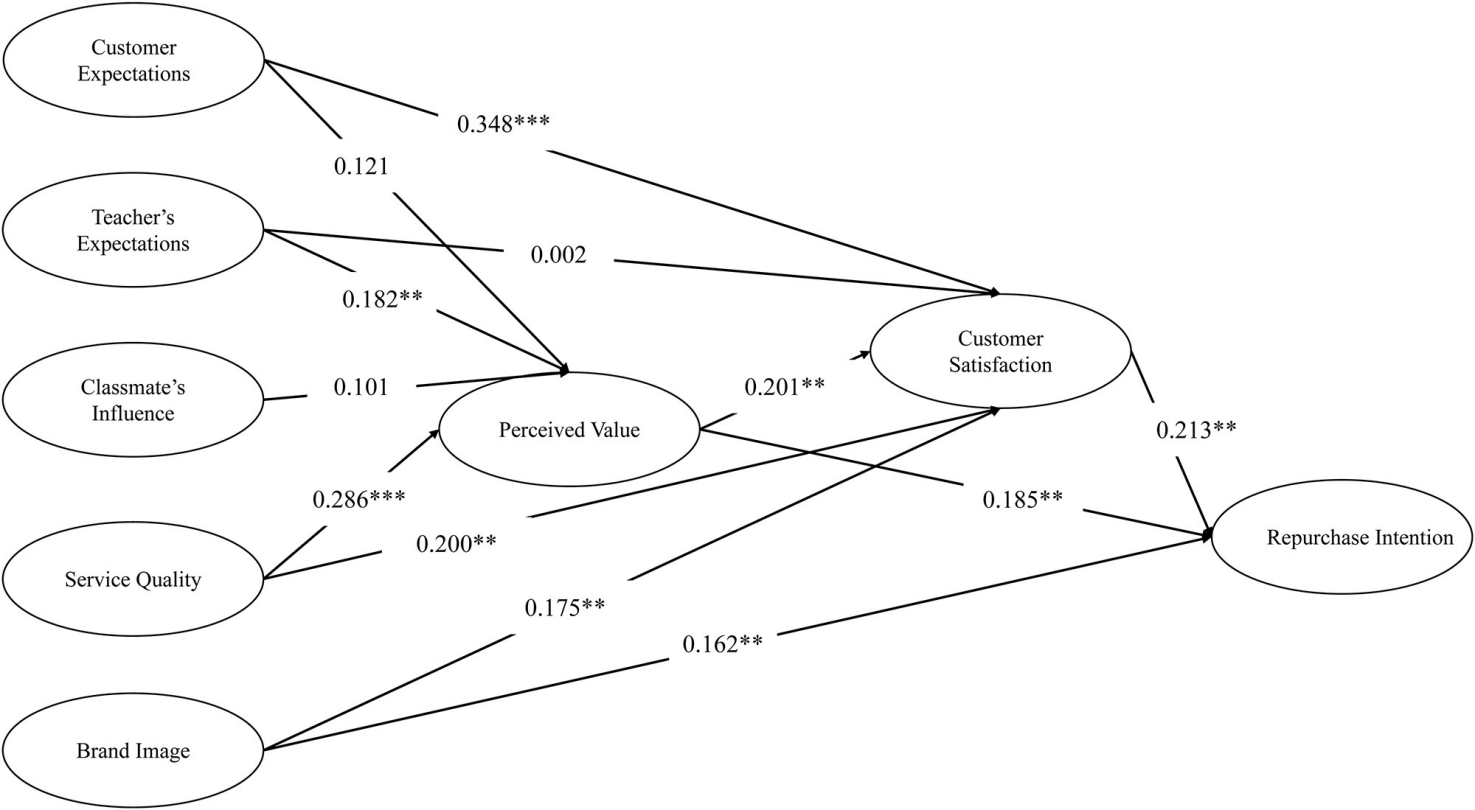
Results of hypotheses testing.

This study investigates the factors influencing customer satisfaction and repurchase intention in music training institutions, utilizing Structural Equation Modeling (SEM) for verification. As shown in Fig 2, a significant positive relationship exists between customer satisfaction and perceived value, brand image, and service quality. This finding supports the Expectation Confirmation Theory, which posits that when customer expectations are met or exceeded, satisfaction and loyalty increase significantly, leading to enhanced repurchase intention. Notably, perceived value and brand image are crucial in driving future purchasing behavior in music training, while service quality remains a vital component for enhancing customer satisfaction. This underscores the necessity for high-quality service in educational settings. Furthermore, the study demonstrates that teacher expectations positively influence perceived value, aligning with the Pygmalion effect. This suggests that teacher trust and expectations can significantly bolster students’ self-efficacy, thereby improving their identification with the course. Although the impact of customer expectations on perceived value was not supported, managing these expectations still plays an important role in shaping overall satisfaction.

This study examines the P-values and path coefficients to evaluate the impact of various factors on customer satisfaction and repurchase intention. The path coefficient for service quality affecting perceived value is 0.333, with a P-value of less than 0.001. This result indicates that service quality significantly positively impacts perceived value, aligning with the SERVQUAL model proposed by AlOmari (2021), which emphasizes the central role of service quality in customer experience [52]. Similarly, the path coefficient for customer expectations on customer satisfaction is 0.319, also with a P-value below 0.001. This supports the Expectation Confirmation Theory articulated by Ozkan et al. (2022), highlighting the significance of customer expectations in the satisfaction formation process [53]. In contrast, the path coefficient for teacher expectations on customer satisfaction is nearly zero, with a high P-value of 0.984, indicating a lack of statistical support. This suggests that teacher expectations are not a primary factor directly influencing student satisfaction in educational services. This finding is consistent with research by Rane et al. (2023), which asserts that customer perceptions and experiences predominantly shape satisfaction [54]. The analysis reveals that larger path coefficients correlate with a more substantial impact on outcome variables, while P-values indicate the statistical significance of these impacts. For educational institutions, high-quality service not only directly enhances students’ perceived value but also indirectly boosts their satisfaction, thereby fostering repurchase intention. Consequently, educational institutions should prioritize improving service quality and managing customer expectations. By fostering interactions and feedback mechanisms between teachers and students, they can enhance the overall educational experience, ultimately increasing student loyalty and satisfaction.

This study offers important insights for the innovation and development of educational institutions, even though some hypotheses were not supported. These insights can help enhance student experience and satisfaction more effectively [55]. First, the lack of significant impact from teacher expectations on customer satisfaction does not diminish the importance of teachers. Educational institutions should reassess teachers’ roles in the learning process and innovate training programs that focus on improving classroom interaction and feedback quality [56]. Positive teacher feedback can significantly boost student motivation and engagement. Institutions could also implement data-driven feedback systems to help teachers adapt their strategies to meet diverse student needs. Second, the low path coefficient for peer influence suggests that interactions among classmates have a limited impact on perceived value. Therefore, institutions should foster cooperation and communication among peers by designing interactive learning activities and team projects [57]. According to Social Learning Theory, positive peer influence can enhance motivation, enabling institutions to build learning communities that encourage sharing of experiences and resources, thus enhancing support and a sense of belonging. Furthermore, the significant role of service quality highlights the need for educational institutions to optimize their services [58]. To improve service quality continuously, educators should establish effective feedback mechanisms. Regular satisfaction surveys can help identify and address issues promptly. Utilizing advanced data analysis tools, institutions can monitor service quality in real-time and respond swiftly to deficiencies. This approach not only enhances perceived value but also increases student loyalty and repurchase intention. Through these strategies, educational institutions can better meet student expectations and enhance the overall educational experience, ultimately achieving sustainable development in a competitive market. However, this study has limitations, including a small sample size and regional constraints. Future research could explore cross-regional comparisons and incorporate qualitative interviews for deeper insights, providing actionable strategies for music education institutions to optimize customer experience and enhance student satisfaction and loyalty.

## 5 Implications

### 5.1 Theoretical implications

This study enhances our understanding of the relationships among customer satisfaction, perceived value, customer expectations, and repurchase intention in the music training market, particularly within music education institutions. It found that customer expectations significantly regulate the relationship between perceived value and customer satisfaction, subsequently influencing repurchase intention. This underscores the crucial role of customer expectations in enhancing customer experience and converting satisfaction into loyalty, aligning with the findings of Japutra et al. (2021) [59], which indicate that customer expectations can improve the attractiveness and retention rates of artistic experiences. Additionally, the positive impact of perceived value on customer satisfaction highlights its importance in fostering satisfaction and loyalty, consistent with Tjoe et al. (2021) [60], which asserts that perceived value enhances emotional engagement and satisfaction. The correlation between customer expectations and perceived value reveals how customers engage with music training content from both cognitive and emotional perspectives, supporting Fa’s (2023) [61] viewpoint on the interaction of cognition and emotion in artistic experiences. Furthermore, the study explored the influence of parental expectations on the emotional experiences of both teachers and customers, confirming that effective instructional design can enhance the transmission of teacher emotions and customer emotional experiences. This research not only broadens the scope of music education theory but also provides practical insights for music training institutions, emphasizing the need to consider customer interaction, emotional experience, and teaching design in developing teaching processes to optimize customer experience and promote cultural inheritance and innovation.

### 5.2 Practical implications

Integrating customer expectations and perceived value into music education and training strategies is essential. Special attention should be given to the diversity of learners’ backgrounds, equipment types, and learning styles. Our findings indicate that music education institutions can enhance learner engagement and effectiveness through thoughtfully designed activities that consider the cultural and language backgrounds of students. For instance, while high-end devices may offer superior learning experiences, it is crucial that all students benefit equally, irrespective of their financial circumstances. Furthermore, successful learning is influenced not only by technical or economic conditions but also by individual efforts, educational resources, and teaching quality. Desianti et al. (2021) found that teaching strategies tailored to customer expectations significantly enhance learning satisfaction [62]. Similarly, Shao et al. (2023) highlighted that perceived value greatly increases students’ emotional engagement [63], while Xia et al. (2023) emphasized the vital connection between teacher emotions and student experiences [64]. Based on these insights, we offer practical recommendations for music education and training aimed at optimizing interaction, emotional experiences, and instructional design. These strategies can enhance educational effectiveness and promote the inheritance and innovation of music culture.

## 6 Conclusion

This study validates the relationships among customer satisfaction, perceived value, customer expectations, and repurchase intention in music training institutions. We employed factor analysis, confirmatory factor analysis, and structural equation modeling to uncover the significant effects of factors such as teacher expectations, service quality, and brand image on customer satisfaction and repeat purchase intention. Our findings confirm that customer satisfaction significantly enhances repurchase intention, with perceived value serving as a crucial driver of customer satisfaction. Customer expectations also play a role by indirectly increasing satisfaction through enhanced perceived value, which further influences purchase intention. However, the direct link between teacher expectations and customer satisfaction is weak, highlighting the necessity of improving the quality of interaction between teachers and students. Furthermore, a strong brand image can effectively boost customer loyalty and guide the strategic positioning of music training institutions in the market. This study provides empirical support for the music training sector and suggests future research should further investigate the relationship between teacher emotions and student satisfaction. Additionally, it should explore strategies to strengthen the connection between students and training institutions, promoting innovation and cultural inheritance in art education.

## Supporting information

S1 Data (XLSX)

S1 Questionnaire (DOCX)
