# Supplementary material for: What drives repurchase retention in music training institutions? examining the roles of customer satisfaction, perceived value, and service quality
Source: PLoS One. 2024 Dec 31;19(12):e0312087. doi: 10.1371/journal.pone.0312087 (PMC11687669; doi:10.1371/journal.pone.0312087)
Supplement: S1 Questionnaire — (DOCX) [file pone.0312087.s002.docx]

| **S/no** | **Construct** | **Items** | **Strongly Agree 5** | **Agree 4** | **Neutral**  **3** | **Disagree**  **2** | **Strongly Disagree**  **1** |
| --- | --- | --- | --- | --- | --- | --- | --- |
| 1 | Customer  Expectations | 在该音乐机构上课已经成为我的习惯。  Taking classes at this music institution has become a habit for me. |  |  |  |  |  |
| 2 |  | 我期望课程的效果很好。  I expect the course to turn out well. |  |  |  |  |  |
| 3 |  | 我期望我的音乐水平得到很大的提升。  I expect my musical skills to improve a lot. |  |  |  |  |  |
| 4 |  | 我期望在该音乐机构得到好的服务。  I expect to receive good service at this music institution. |  |  |  |  |  |
| 1 | Teacher’s  Expectations | 音乐机构的声乐课程的教学安排很丰富。  The teaching schedule of vocal courses in music institutions is rich. |  |  |  |  |  |
| 2 |  | 学生很快就喜欢上授课老师。  Students quickly like their teachers. |  |  |  |  |  |
| 3 |  | 该音乐机构的声乐课程课堂气氛好，教学效果明显。  The vocal music courses at this music institution have a good classroom atmosphere and obvious teaching effects. |  |  |  |  |  |
| 1 | Classmate’s  Influence | 我看到别人报名该音乐机构的课程，我也想报名。  I saw others signing up for classes at this music institution and I wanted to sign up too. |  |  |  |  |  |
| 2 |  | 当同学的成绩提升时，我也会努力。  When my classmates' grades improve, I will work harder. |  |  |  |  |  |
| 3 |  | 当同学的成绩下降时，我也会懈怠。  When my classmates' grades dropped, I would slack off. |  |  |  |  |  |
| 4 |  | 我会受到同学的干扰。  I will be disturbed by my classmates. |  |  |  |  |  |
| 5 |  | 同学会影响我在该音乐机构续费。  The classmate association will affect my renewal of membership at this music institution. |  |  |  |  |  |
| 1 | Service  Quality | 该音乐机构的工作人员给我的第一印象很好。  My first impression was very good with the staff at this music institution. |  |  |  |  |  |
| 2 |  | 该音乐机构声乐课程的授课老师很专业。  The teachers who teach voice courses at this music institution are very professional. |  |  |  |  |  |
| 3 |  | 该音乐机构声乐课程的教学反馈很及时。  The teaching feedback of the vocal courses of this music institution is very timely. |  |  |  |  |  |
| 4 |  | 该音乐机构干净整洁，有隔音措施，乐器品质优良。  The music institution is clean, soundproofed and the instruments are of high quality. |  |  |  |  |  |
| 1 | Brand  Image | 该音乐机构品牌口碑好。  The brand of this music institution has a good reputation. |  |  |  |  |  |
| 2 |  | 仅从该品牌的服装，我就可认出该品牌。  I recognize this brand just from its clothing. |  |  |  |  |  |
| 3 |  | 我很喜欢该音乐机构的课程效果。  I really like the effectiveness of the courses at this music institution. |  |  |  |  |  |
| 4 |  | 该音乐机构环境舒适。  The music institution has a comfortable environment. |  |  |  |  |  |
| 5 |  | 该品牌机构知名度很高。  The brand agency is well known. |  |  |  |  |  |
| 1 | Perceived  Value | 相较于该音乐机构的声乐课程所学到的知识，我认为购买这门课程是值得的。  Compared to what I learned from the vocal course at this music institution, I think it was worth purchasing this course. |  |  |  |  |  |
| 2 |  | 相较于该音乐机构的声乐课程所得到的收获，我认为花费的时间和精力是值得的。  Compared to what I get from the vocal lessons at this music institution, I think the time and effort spent is worth it. |  |  |  |  |  |
| 3 |  | 我认为通过该音乐机构的声乐课程对我来说有很高的价值。  I think the voice lessons through this music institution were of high value to me. |  |  |  |  |  |
| 4 |  | 我会在该音乐机构续费，提升我的音乐水平。  I will renew my membership at this music institution and improve my music skills. |  |  |  |  |  |
| 1 | Customer  Satisfaction | 我对该音乐机构的声乐课程的总体感觉很满意。  I am generally satisfied with my overall feeling about the vocal program at this music institution. |  |  |  |  |  |
| 2 |  | 我对该音乐机构的声乐课程的总体感觉很愉快。  My overall feeling about the vocal program at this music institution was positive. |  |  |  |  |  |
| 3 |  | 该音乐机构的声乐课程能满足我的学习需求。  The music institution's vocal lessons meet my learning needs. |  |  |  |  |  |
| 1 | Repurchase Intention | 以后我会继续在该音乐机构上课。  I will continue to take classes at this music institution in the future. |  |  |  |  |  |
| 2 |  | 我很乐意长期在该音乐机构上课。  I would love to take classes at this music institution on a long-term basis. |  |  |  |  |  |
| 3 |  | 我愿意向身边的人推荐该音乐机构。  I would recommend this music institution to everyone around me. |  |  |  |  |  |
